# Experiences, perceptions and preferences of mothers towards childhood immunization reminder/recall in Ibadan, Nigeria: a cross-sectional study

**DOI:** 10.11604/pamj.2015.20.243.6019

**Published:** 2015-03-13

**Authors:** Victoria Bolanle Brown, Abimbola Oluwatosin, Martins Olusola Ogundeji

**Affiliations:** 1 School of Nursing, University College Hospital, Ibadan Nigeria; 2 Department of Nursing, Faculty of Clinical Sciences, College of Medicine, University of Ibadan, Nigeria; 3 Primary Health Care and Health Management Centre (PriHEMAC), Ibadan, Nigeria

**Keywords:** Mothers, experiences, preferences, childhood, immunization, reminder/recall, community

## Abstract

**Introduction:**

Immunization reminder/recall system is proven as one of the effective ways of improving immunization rates. Prior to the development and implementation of an immunization reminder/recall system intervention, we explored the experiences, preferences and perceptions towards childhood immunization reminder/recall among 614 mothers of infants in Ibadan, Nigeria.

**Methods:**

A cross-sectional health facility-based survey utilizing a semi-structured questionnaire was conducted in four Primary Health Care centers. Descriptive statistics were computed using SPSS. Logistic models were used to investigate the relationships with specific outcomes.

**Results:**

Only 3.9% had ever heard of immunization reminder/recall and 1.5% had ever received one. However, 97.9% were willing to record their cellphone numbers in the clinics for immunization reminder/recall and 95.1% were willing to receive. Their preferred communication modes were cell phone calls (57.6%) or text messages/SMS (35.6%). Only 2.2% preferred home-visits and 0.4%, e-mails. About 4% were not willing to receive any form of immunization reminder/recall. Mothers with post-secondary education were more likely to prefer SMS than other mothers (OR 2.3, 95% CI 1.7-3.3, p.

**Conclusion:**

This study provided critical baseline data for designing a reminder/recall intervention for routine childhood immunization in the study communities. The findings may serve as a guide for public health professionals in designing reminder/recall strategies to improve childhood immunization.

## Introduction

Immunization is one of the most powerful and cost effective of all public health interventions [1]. Immunization prevents debilitating illness and disability and saves millions of lives every year. It is also key to achieving Millennium Development Goal Four (MDG 4) [1]. Despite the evidence-based successes in reducing vaccine-preventable diseases (VPDs) morbidity and mortality, routine childhood immunization compliance in Nigeria is suboptimal. The immunization completion rate was 10% among children aged 9-12 months and 53% among children aged 12-23 months according to the Nigeria National Immunization Coverage Survey (NICS) conducted in 2010 [2]. This rate is well below the 90% level recommended by the World Health Organization (WHO) for the sustained control of VPDs. Globally, vaccine preventable diseases account for nearly 20% of all deaths occurring annually among children under five years of age [3]. The 2010 global routine vaccination coverage showed that about 19.3 million children remained at risk for diphtheria, tetanus, and pertussis and other vaccine-preventable causes of morbidity and mortality, and about 50% of these children are from India, Nigeria, and Congo [4]. Nigeria´s routine immunization schedule requires that infants be vaccinated with the following vaccines: a dose of Bacillus Calmette-Guerin (BCG) vaccine at birth (or the first week of life); three doses of diphtheria, pertussis and tetanus (DPT) vaccine at 6, 10 and 14 weeks of age; at least four doses of oral polio vaccine (OPV) - at birth, 6, 10 and 14 weeks of age; and one dose of measles vaccine at nine months of age. In 2004, hepatitis B and yellow fever vaccines were included in the country's schedule, recommending the receipt of three doses of hepatitis B (hepB) vaccine at birth, 6 and 14 weeks of age while yellow fever vaccine should be given at nine months of age, along with measles vaccine. In May 2012, the Nigerian government introduced the pentavalent vaccine, which is a combination of five vaccines-in-one that prevents diphtheria, tetanus, whooping cough, hepatitis b and haemophilus influenza type b, in a single dose [5]. Instead of children taking different vaccines at different times, the pentavalent vaccine is administered in three doses between the ages of 6 weeks and 14 weeks at an interval of 4 weeks. The pentavalent vaccine replaced the DPT vaccine administered at 6, 10, and 14 weeks and HBV administered at birth, 6 and 14 weeks. With the introduction of the pentavalent vaccine Nigeria's expanded program on immunization now has a new schedule as follows: antigens BCG, OPV0, hepB0 (at birth) OPV (3 doses), pentavalent (3 doses), measles and yellow fever.

Vaccines are given at recommended scheduled intervals. The timing and spacing of vaccine doses are two of the most important issues in the appropriate use of vaccines [6]. Studies have shown that the right doses at the right interval through the right route generate the optimal immune response [7, 8]. Untimely receipt of immunization can expose children to the risk of VPDs with their associated morbidity and mortality [9, 10]. The use of reminder/recall (RR) systems is viewed as an effective way to improve adherence to recommended immunization schedules [11–14]. A “reminder” is the postcard, letter or telephone call reminding clients of immunizations before they are due and prompting them to return to the clinic to receive the recommended immunizations. A “recall” is the postcard, letter or telephone call after clients miss an appointment or when an individual has fallen behind on scheduled immunizations. The recall prompts clients to return to the clinic to catch up on needed immunizations [15]. Use of mobile phone technology to aid clients’ compliance with and adherence to healthcare guidelines represents an advance in public health care delivery system, especially in developed countries. Though Nigeria is a developing country, the use of mobile phone technology is particularly high. The Central Intelligence Agency (CIA) World Factbook ranked the country as the 10th highest country in cell phone usage out of 217 countries globally. According to the factbook, Nigeria had about 112.78 million cellular phone subscribers in the year 2012 [16]. This information is corroborated by the report of the National Bureau of Statistics in Nigeria for the year 2012, which showed 68.49% of teledensity in Nigeria and that the use of cell phones is increasing in all age groups [17]. This information on cellular phone usage provides a strong platform from which to explore the adoption of its use to improve immunization compliance. Few studies have examined mothers’ experiences, preferences and perceptions towards receiving the childhood immunization reminder/recall (IRR) in Nigeria. This baseline survey was conducted among mothers of infants in four communities in Ibadan, southwest Nigeria to obtain a better understanding of this subject matter prior to the development and implementation of the immunization reminder/recall system intervention in the study communities. The aim of this study was to explore mothers’ experiences, preferences and perceptions towards receiving the childhood immunization reminder/recall.

## Methods

**Study design:** a descriptive cross-sectional health-facility-based survey.

**Study population** participants were 614 mothers of infants attending routine immunization clinics in four randomly selected communities’ PHC centers in Ibadan, Nigeria. Eligibility criteria were being a mother of a child aged 0-12 weeks and living within the study community. After obtaining informed consents, the survey interview was conducted with 614 willing and eligible mothers of children enrolled in a larger study that evaluated the effects of a community health nurse-led intervention on childhood immunization completion in the study communities.

**Data collection:** ethical approval was obtained for the main study from the Oyo State Research Ethical Review Committee (Approval Number: AD 13/479/209) before the commencement of the data collection. After giving detailed description of the research, consent for participation in the study was taken from the mothers who met the inclusion criteria. The survey data were collected during childhood routine immunization clinic sessions over a period of 15 weeks between August and November 2012. The data collection involved the use of a semi-structured questionnaire from which items relevant for this paper were extracted. The questionnaire was designed on the basis of an extensive review of the literature and experts’ opinions. It was translated from English into the local language (Yoruba), then back to English and pilot-tested with other instruments for the main study. The items that explored the experiences, preferences and perceptions of mothers towards reminder/recall were analyzed. The items included: questions on socio-demographic characteristics of the participants; seven items that assessed mothers’ prior experiences of IRR and willingness to receive IRR; three items that assessed their preferences for IRR and 15 items on a 5-points Likert scale that explored their perceptions about IRR. After obtaining consent, the questionnaires were administered by five trained interviewers in either English or the local language and each entire survey took about 20 minutes to be administered.

**Data analysis:** data entry and analysis were carried out using the SPSS Version 21 software (IBM Corporation, Armonk, NY). Descriptive statistics were computed to describe participants’ socio-demographic characteristics. Continuous variables were summarized using means and standard deviations (SD). Mothers’ preferences for reminder/recall were presented as frequencies and contingency tables as well as bar chats as appropriate. The responses to the various reminder/recall perceptions statements were dichotomized as “agreed” and “disagreed” Logistic regression models were used to investigate factors associated with mothers’ experiences, preferences and perceptions of IRR. Odds ratios (OR) and 95% confidence intervals (CI) were computed for each predictor variable. P-values

## Results

**Socio-demographic characteristics of participated mothers:** the age of the participants ranged from 15 to 42 years with mean age 29 (SD 4.9) years. Almost all (98.7%) were married to their infants’ fathers and 45.9% completed secondary education. The socio-demographic characteristics of the participated mothers are shown in Table 1.

**Table 1:** Socio-demographic characteristics of the mothers studied

Socio-demographic Characteristics	Frequency	Percent (%)
**Age**		
15-19	10	1.6
20-24	109	17.8
25-29	207	33.7
30-34	206	33.6
35-39	72	11.7
40-42	10	1.6
Total	614	100
**Marital Status**		
Married to child's father	606	98.6
Separated from child's father	1	0.2
Never married	6	1.0
Widowed	1	0.2
Total	614	100
**Number of Children**		
1	203	33.1
2	195	31.7
3	124	20.1
4	68	11.1
≥5	24	4.0
Total	614	100
**Level of Education**		
No formal education	5	0.8
Some primary education	8	1.3
Completed primary education	50	8.1
Some secondary education	33	5.4
Completed secondary education	282	45.9
Post-secondary education	236	38.5
Total	614	100

**Mothers’ experiences of and preferences for childhood immunization reminder/recall:** six hundred and seven mothers (98.9%) of the 614 either possessed at least a cell phone with a valid number each or the spouse possessed at least one. Only 24 (3.9%) of them had ever heard of immunization reminder/recall and nine (1.5%) had ever received one. However, 601 (97.9%) were willing to record their cellphone numbers at the immunization clinics for reminder while 584 (95.1%) were willing to receive reminder/recall information about their children immunization. As shown in Table 2, phone calls and text messages/SMS were their leading preferences for communicating immunization reminder/recall, only few preferred home-visits, letters and e-mails.

**Table 2:** Mothers’ preferred modes of communication about immunization reminder/recall

Reminder/recall mode preferred	Frequency	Percent (%)
Letters and e-mails	3	0.4
Cellphone calls	415	57.6
Text messages / SMS	257	35.5
Home visits	16	2.2
Do not want to receive immunization reminder/recall	30	4.2

Multiple responses

**Figure 1:**
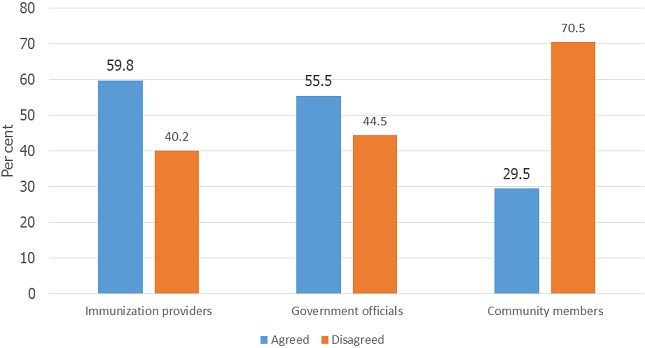
Mothers’ preferred senders of childhood immunization reminder/recall

**Figure 2:**
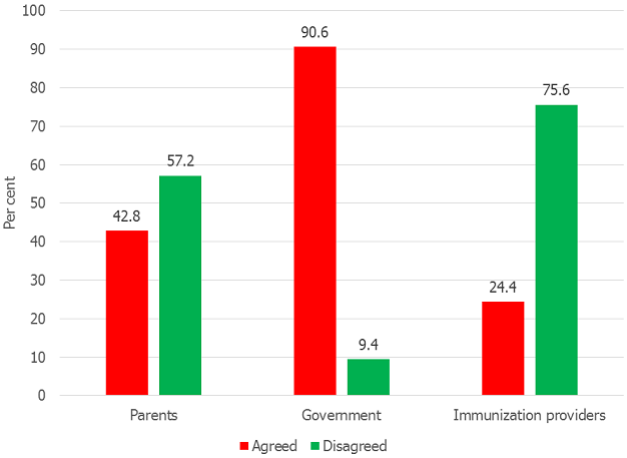
Mothers’ preferred sources of funding for childhood immunization reminder/recall

**Mothers’ preferences for immunization reminder/recall senders and sources of funding:** almost 60% of the mothers agreed that the immunization providers should be those that will send the IRR to them. Concerning funding for IRR, most of them (90.6%) were of the opinion that government should pay for immunization reminder/recall, although many (42.8%) were also willing to bear the cost (Figure 1 and Figure 2).

**Mothers perceptions about childhood immunization reminder recall:** five hundred and eighty (95.6%) in the study believed that adherence to immunization schedule is important. Although 374 (60.9%) were of the opinion that mothers should not forget their children immunization appointments, 570 (92.8%) still believed that it is important for parents to be reminded of their children immunization before the appointment day. Almost all the mothers (606) (98.7%) perceived that immunization reminders will be helpful to mothers in complying with their children immunization schedules.

**Factors associated with preferences and perceptions of mothers towards childhood immunization reminder/ recall:** Table 3 shows the factors associated with mothers’ experiences, preferences and perceptions towards childhood immunization reminder/ recall. As revealed in the table, mothers with post-secondary education were twice as likely as other mothers to prefer text messages/SMS (OR 2.3, 95% CI 1.7-3.3), but only half were as likely to prefer phone calls (OR 0.5, CI 0.32-1.0). Maternal age was not a significant predictor of preference for cell phone calls (OR 1.0, CI 0.97-1.04, p = 0.660) reminder/recall or SMS (OR 1.0, 95% CI 0.98-1.05).

**Table 3:** Logistic model showing the association between maternal age and education with preferred mode of receiving immunization reminders/recall

Preferred mode	Maternal Age			Maternal Education		
	OR (SE)	95% CI	p	OR (SE)	95% CI	p
Phone call	1.003 (0.021)	0.97-1.04	0.888	0.5 (0.092)	0.32-1.0	<0.0001*
SMS	1.016 (0.019)	0.98-1.04	0.419	2.318 (0.445)	1.7-3.3	<0.0001*

* Statistically significant

**Association between maternal age and education on preferred source of receiving immunization reminders/recall:** The association between maternal age and education on preferred source of receiving immunization reminders/recall is shown in Table 4. There is no significant association between maternal age and preferred source of receiving immunization reminders/recall (p > 0.05). Also no significant association was found between maternal education and preferred source of receiving immunization reminders/recall (p > 0.05).

**Table 4:** Logistic model showing the association between maternal age and education on preferred source of receiving immunization reminders/recall

Preferred source	Maternal Age			Maternal Education		
	OR (SE)	95%CI	p	OR (SE)	95% CI	p
Providers	1.030 (0.019)	0.99-1.05	0.118	1.093 (0.210)	0.81-1.52	0.643
Health workers	1.016 (0.019)	0.98-1.05	0.400	1.297 (0.252)	0.97-1.93	0.181
Government	1.025 (0.019)	0.98-1.05	0.183	1.233 (0.234)	0.81-1.57	0.269
Community	1.029 (0.021)	0.99-1.07	0.165	0.893 (0.186)	0.64-1.32	0.588

**Association between maternal age and education and preferred source of funding for immunization reminders/recall:** As demonstrated in Table 5, no significant association was found between maternal age and their preferred source of funding for immunization reminders/recall (p > 0.05). However, a significant association was found between maternal education and preferred source of funding for immunization reminders/recall; mothers with post-secondary education were as twice as likely to prefer government as the source of funding for immunization reminders/recall to other sources of funding (OR 2.218, 95% CI 1.02-3.49).

**Table 5:** Logistic model showing the association between maternal age and education and preferred source of funding for immunization reminders/recall

Preferred source of fund for immunization reminder/recall	Maternal Age			Maternal Education		
	OR (SE)	95% CI	p	OR (SE)	95% CI	p
Parents	1.035 (0.020)	0.99-1.06	0.066	0.752 (0.145)	0.48-0.94	0.138
Providers	1.001 (0.022)	0.95-1.03	0.974	0.861 (0.191)	0.55-1.20	0.498
Government	0.994 (0.031)	0.95-1.06	0.853	2.218 (0.832)	1.02-3.49	0.034*

* Statistically significant

## Discussion

The findings of this study demonstrate mothers’ support of reminder/recall for routine childhood immunization at primary health care setting. Although very few of them have had experience with immunization reminder/recall, access to cellphones by the majority of the mothers, their willingness to record their cellphone numbers at the immunization clinics and to receive immunization reminder/recall indicates the feasibility of implementing immunization reminder/recall in the study communities. Mothers’ willingness to receive immunization reminder/recall corroborates the findings in Lagos and Benin in Nigeria [18, 19]. The findings reveal that the mothers’ most preferred reminder/recall mode is cellphone call followed by short message service (SMS). This preference may be attributed to their access to cellphones more than letters and emails. Previous studies in New York City and Kansas in USA have revealed a wide support and acceptability for text messages / short message service (SMS) as a mode of immunization reminder/recall [20–23]. Also, person to person telephone reminders has also been found to be preferred by parents in studies in Nigeria and USA [18, 24]. It is possible that mothers who preferred cellphone call reminders in this study may have done so because they are likely to have the opportunity to express themselves if they plan to attend their children schedule immunization clinic or request to change appointment date if they cannot attend for any reason [25]. In this study, most mothers preferred the immunization providers/health workers to contact them during immunization reminder/recall even though some do not mind government officials or community members to make the contact. The majority that preferred immunization providers/health workers to contact them may see it as a demonstration of a better relationship between the health workers and clients. This indicates the importance of establishing a strong and trusting nurse/client relationship by community health nurses in other to effectively communicate immunization information. Mothers in this study may also see reminder/recall as a means by which the immunization providers reinforcing mothers’ compliance with their children immunization. More research needs to be undertaken to explore reasons for mothers’ preference for IRR in future studies.

It is not surprising to find out in this study that majority of mothers agreed with the idea of the government paying for immunization reminder recall. This may be because they are used to the government providing free immunization services in Nigeria. Mothers’ agreement with this idea supports previous research in Lagos, Nigeria [18]. This finding has implication for public health program planners to include the cost of reminder/recall as part government financial responsibility towards routine childhood immunization. However, some mothers in this study also indicated their willingness to pay, probably because they value their children's health and believe that parents are supposed to pay to protect their children's health. The perception of the mothers in the study demonstrates their support for childhood immunization because over half of them are of the opinion that mothers should not forget their children immunization appointment days. Despite this they still support the use of immunization reminder/recall. The findings show that mothers’ education was associated with their preference for communication mode for reminder/recall. Mothers with post-secondary education opted for text messages/SMS. This result may be explained by the fact that higher education may facilitate the use and the ability to read SMS. Mothers with tertiary education are likely to be aware of incoming SMS and are likely to read and act on the message promptly. Advocating for female education should be of paramount importance to community health nurses as education has been found to influence the ways women receive health information and their attitude towards the information [26]. The findings on preference for SMS in this current study are consistent with findings in Lagos, Nigeria [18]. Answering a phone call may not demand as much level of intelligence as reading SMS, this may make receiving a call more acceptable for less educated women. Meanwhile, maternal age was not a significant predictor of preference for cell phone calls or SMS in the current study. However, findings from previous study in USA showed that parents aged 30 years and above preferred e-mail for reminder [24].

**Limitations:** this study has some limitations. This survey was conducted in urban and sub-urban community health facilities and no rural community was involved. The findings may therefore not be generalizable to all populations.

**Recommendations for future research:** The results from this study suggest mothers’ willingness to receive reminder/recall for childhood immunization. Findings also indicate that mothers prefer reminder/recall information to be sent by immunization providers/health care workers. For future research, it is pertinent to explore immunization providers’ attitudes towards sending reminder/recall to improve childhood routine immunization.

**Implications for community health nurses and other public health professionals:** in designing reminder/recall strategy to improve childhood immunization uptake, community health nurses and other public health professionals need to consider mothers’ preferences and perceptions about the strategy before the implementation as “one-size-fits-all” approach in the mode of sending immunization reminder/recall to mothers may have less impact. It may also be necessary for public health professionals in collaboration with public health policy makers to organize trials for implementation of the strategy in a larger scale in PHC facilities to work out the logistics and the effectiveness.

## Conclusion

Mothers in Ibadan, southwest Nigeria had little experience about immunization reminder/recall system, but they were willing to receive immunization reminder/recall for their children immunization appointment. Their preferred modes of reminder/recall were cellphone and SMS. Public health professionals may find the information from this study useful in the designing and implementation of reminder/recall interventions for childhood immunization. However, keeping accurate and complete immunization records is important for effective reminder/recall activities.
